# Supplementary material for: The nucleocytoplasmic translocation of HINT1 regulates the maturation of cell density
Source: Life Sci Alliance. 2025 Jul 22;8(10):e202503215. doi: 10.26508/lsa.202503215 (PMC12284375; doi:10.26508/lsa.202503215)
Supplement: Supplementary file 1 [file LSA-2025-03215_SdataF1_F7.docx]

**Source data for unprocessed blots for Fig. 1C, 7A, 7B and 7C. Only the lanes outlined in red pertain to this paper.**

**Fig. 1C:**


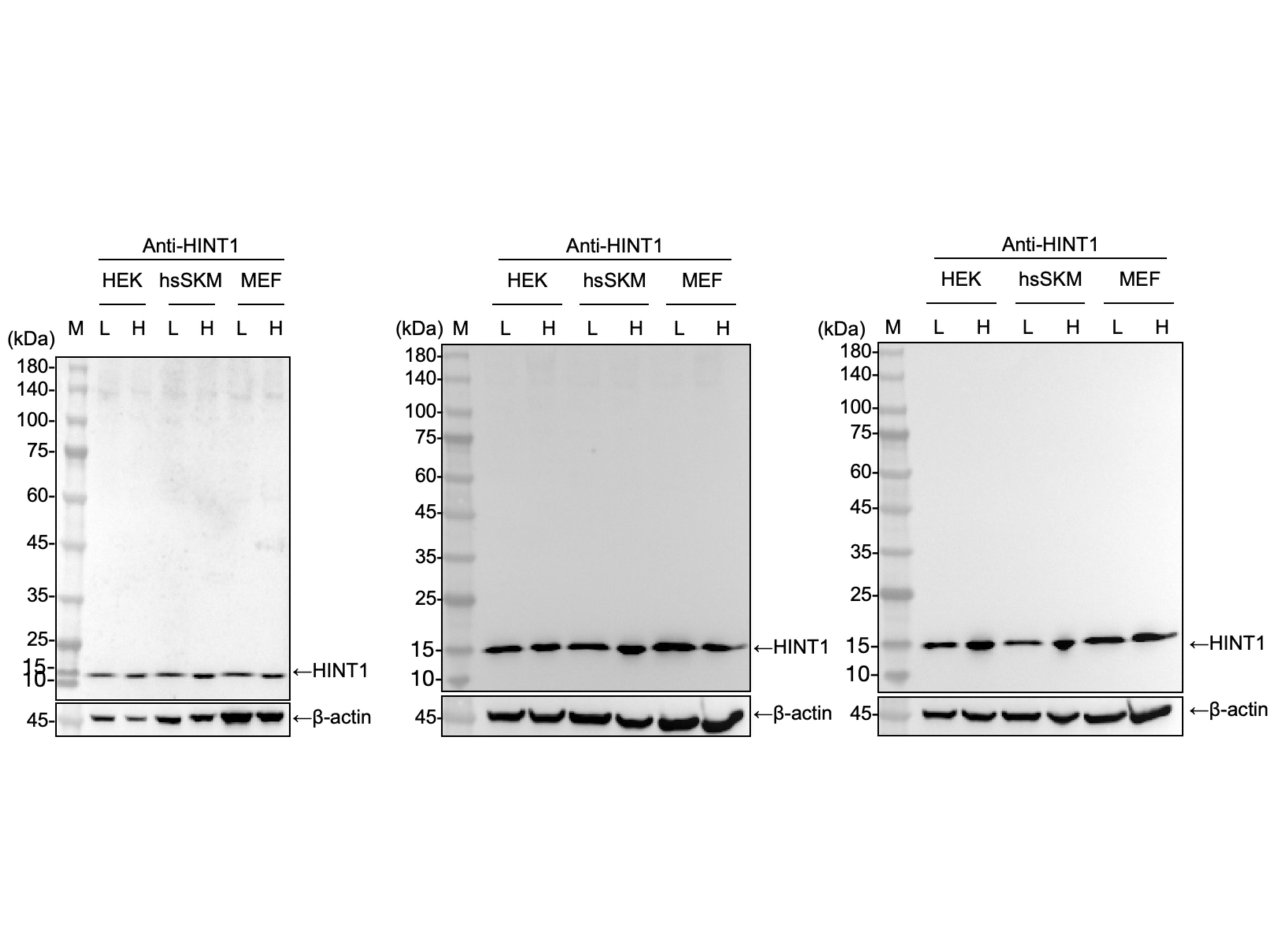


**Fig. 7A:**


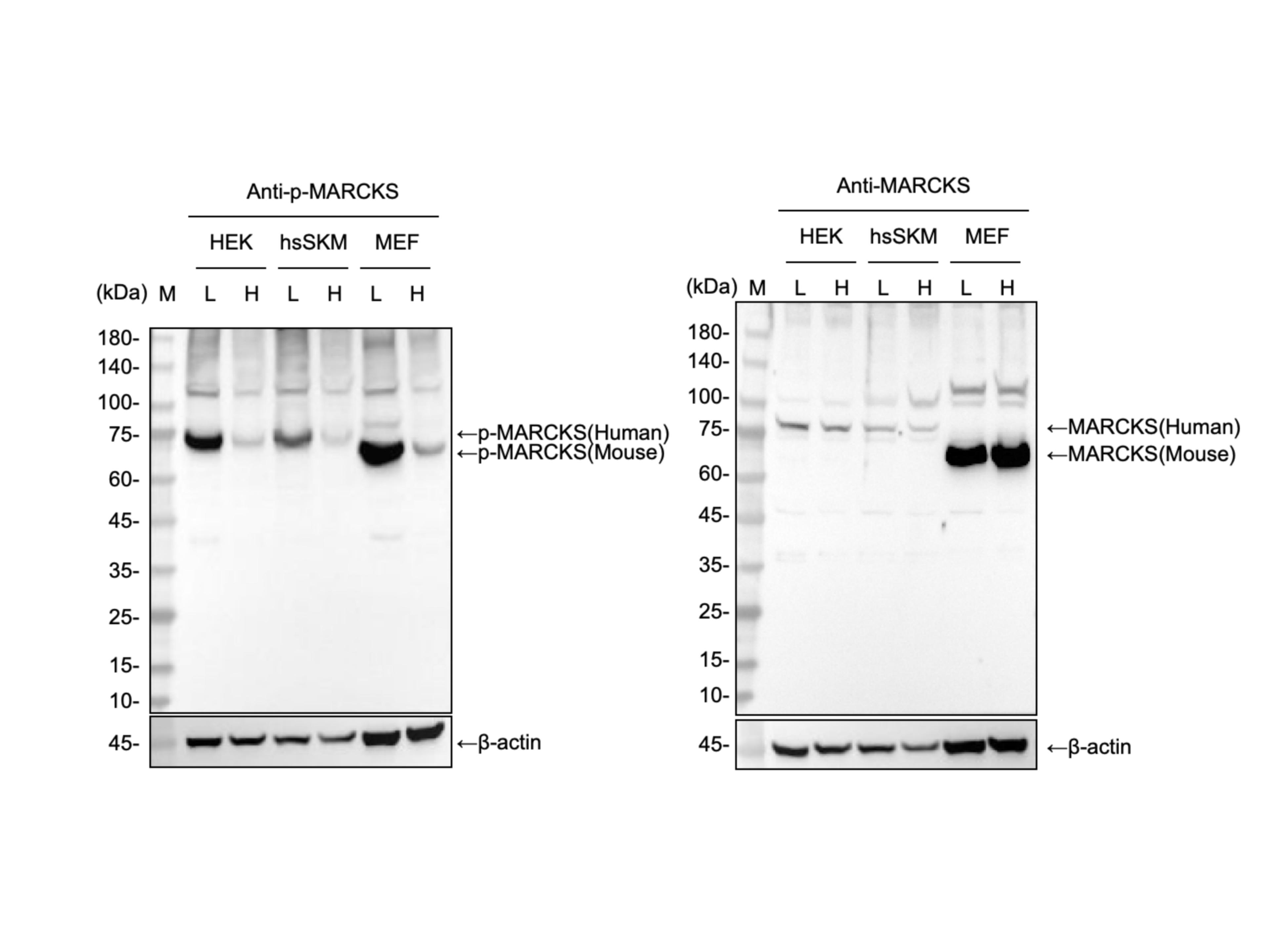


**Fig. 7B:**

**
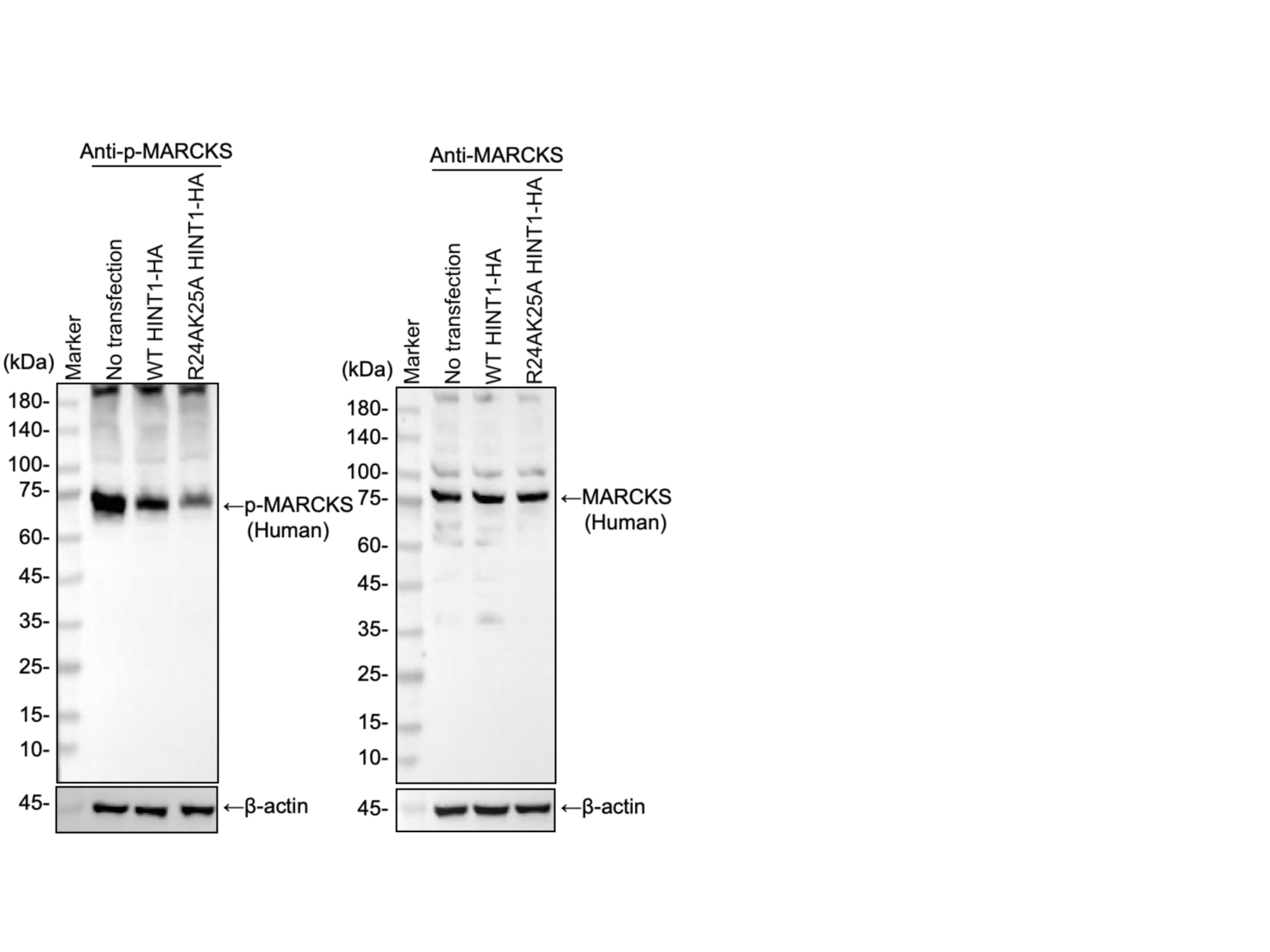
**

**Fig. 7C:**

**
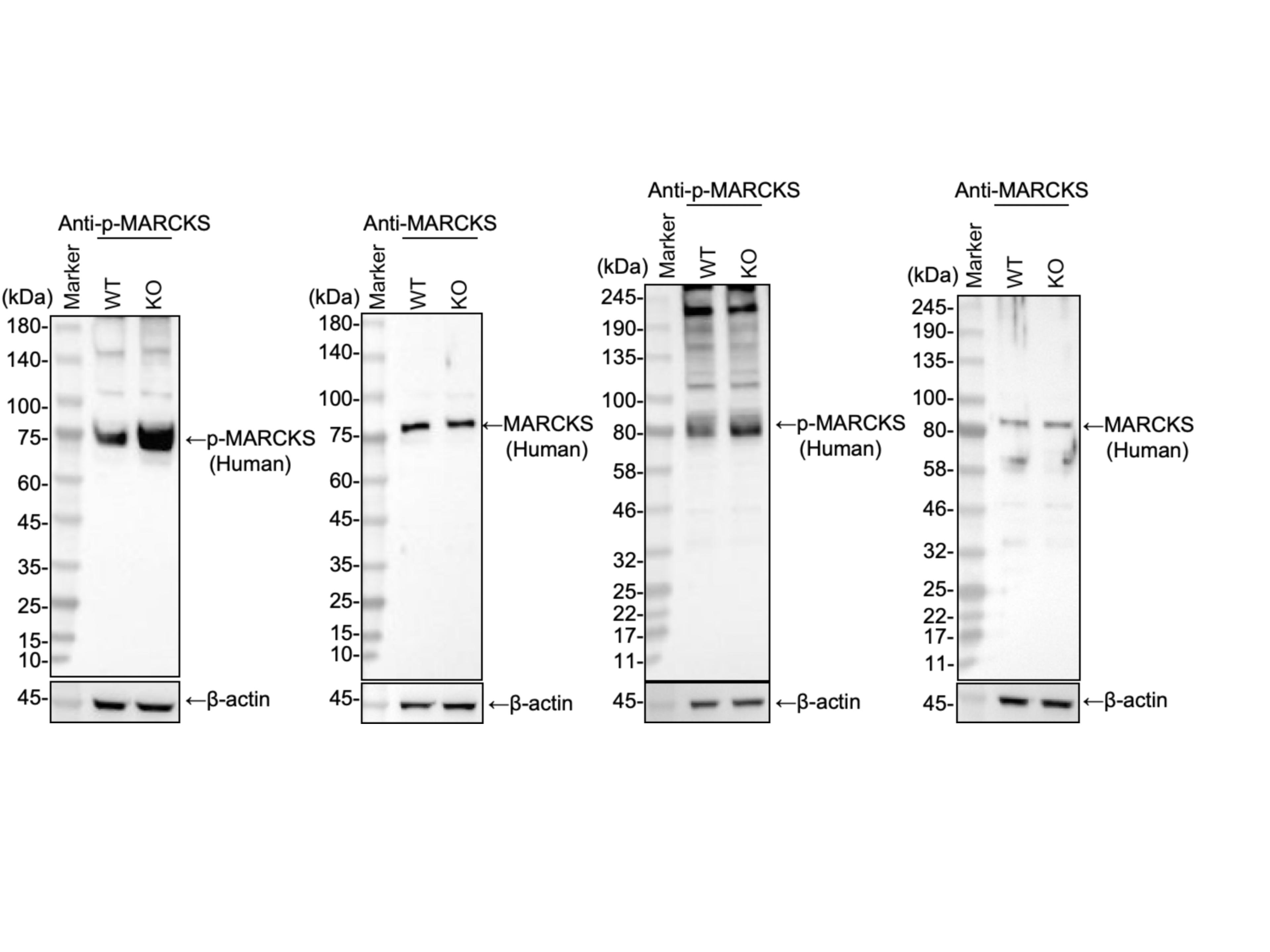
**
